# Effects of Tea Saponin Supplementation on Nutrient Digestibility, Methanogenesis, and Ruminal Microbial Flora in Dorper Crossbred Ewe

**DOI:** 10.3390/ani9010029

**Published:** 2019-01-21

**Authors:** Yunlong Liu, Tao Ma, Dandan Chen, Naifeng Zhang, Bingwen Si, Kaidong Deng, Yan Tu, Qiyu Diao

**Affiliations:** 1 Key Laboratory of Feed Biotechnology of the Ministry of Agriculture, Feed Research Institute, Chinese Academy of Agricultural Sciences, Beijing 100081, China; liuyunlong9438@163.com (Y.L.); matao@caas.cn (T.M.); chendancd@126.com (D.C.); zhangnaifeng@caas.cn (N.Z.); sibingwen@caas.cn (B.S.); 2 College of Animal Science, Jinling Institute of Technology, Nanjing 210000, China; kdeng3@yeah.net

**Keywords:** methane emissions, nitrogen balance, reduction strategy, rumen fermentation, microbial flora, tea saponins

## Abstract

**Simple Summary:**

Greenhouse gas emissions are a serious cause of global warming and climate change, and have become a common focus for all countries. Methane has been proven the second most commonly occurring greenhouse gas. Ruminants have been blamed for substantially contributing to methane emissions. Supplementation with tea saponin (TS) effectively decreased methane emissions and nitrogen emissions. It is not only beneficial for environmental protection, but also has potential economic benefits.

**Abstract:**

Two experiments were conducted using Dorper × thin-tailed Han crossbred ewes. In experiment 1, eighteen ewes were randomly assigned to two dietary treatments (a basal diet, or the same basal diet supplemented with 2.0 g tea saponin (TS)/head/day) to investigate the effects of TS supplementation on nutrient digestibility and methane emissions. In experiment 2, six ewes with ruminal cannulae were assigned to the same two dietary treatments as in experiment 1 to investigate the effects of TS supplementation on rumen fermentation and microbial flora. TS supplementation increased the apparent digestibility of organic matter (OM) (*p* = 0.001), nitrogen (N) (*p* = 0.036), neutral detergent fibre (NDF) (*p* = 0.001), and acid detergent fibre (ADF) (*p* < 0.001). Urinary N (*p* = 0.001) and fecal N (*p* = 0.036) output were reduced, and N retention (*p* = 0.001) and nitrogen retention/nitrogen intake (*p* = 0.001) were increased. Supplementary TS did not decrease absolute methane emissions (*p* = 0.519) but decreased methane emissions scaled to metabolic bodyweight by 8.80% (*p* = 0.006). Ammonia levels decreased (*p* < 0.001) and total volatile fatty acid levels increased (*p* = 0.018) in response to TS supplementation. The molar proportion of propionate increased (*p* = 0.007), whereas the acetate:propionate ratio decreased (*p* = 0.035). Supplementation with TS increased the population of *Fibrobacter succinogenes* (*p* = 0.019), but the population of protozoans tended to decrease (*p* = 0.054). Supplementation with TS effectively enhanced the apparent digestibility of OM, N, NDF, and ADF, and decreased methane emissions scaled to metabolic bodyweight.

## 1. Introduction

Greenhouse gas emissions are a serious cause of global warming and climate change, and have become a common focus for all countries, according to the Intergovernmental Panel on Climate Change [1]. Methane is the second most important anthropogenic greenhouse gas, which has 21 times the global warming potential of carbon dioxide [2]. Agriculture accounts for approximately 40% of the total methane emissions from anthropogenic sources, with 25% coming from enteric fermentation in livestock [3]. In ruminants, approximately 95.5% of the methane is produced by feed fermentation in the rumen [4] and is exhaled through the nose and mouth; it represents a loss of 2–12% of the feed energy, depending on the diet [5]. Consequently, numerous efforts are underway to manipulate rumen fermentation and the rumen microbial ecosystem to reduce methane emissions. Limiting the methane emissions from ruminants is not only beneficial for environmental protection, but also has potential economic benefits [6].

Many chemical feed additives have been used to inhibit methane emissions, but these additives are either toxic to the hosts or only have a transient effect on methanogenesis [7]. By contrast, plant extracts are attractive as additives for animal feeds and animal health agents, as they are considered natural, safe, and efficient, and have no hormonal consequences or negative side effects [8]. One promising plant compound is tea saponin (TS), which is a class of pentacyclic triterpenoid glucoside compounds found in a variety of tea plants (Camelliaceae). The basic structure consists of ligands, sugars, and organic acids. Tea saponin has been reported to have an inhibitory effect on protozoa by affecting cell membrane integrity. As protozoa are known to be positively correlated with methanogenesis, tea saponin’s biological properties can be used to suppress methane production [9], reduce rumen protozoan counts, and modulate rumen fermentation patterns [10,11]. 

The rumen is a fermentation chamber where a large number of microbes, including bacteria, protozoa, and fungi, coexist and conduct complicated fermentation processes. Previous studies have been carried out on the effect of TS on methane emissions [12,13,14]; however, most trials were carried out in vitro [9,15], so the results do not necessarily reflect the situation in vivo [16]. Consequently, the mechanism of action of TS remains unclear. The aim of the present study was to investigate the effects of dietary TS supplementation on ruminal fermentation characteristics, digestibility, methanogenesis, and the ruminal microbial flora using sheep as in vivo model. We hypothesized that TS supplementation could reduce methane emissions by inhibiting the growth of ruminal methanogens and protozoa, and may have different effects on cellulolytic bacteria.

## 2. Materials and Methods 

This study was conducted from March to May 2013 at the Experimental Station of the Chinese Academy of Agricultural Science (CAAS), Beijing, China. The experimental procedures were approved by the Animal Ethics Committee of the CAAS, and humane animal care and handling procedures were followed throughout the experiment (protocol number: AEC-CAAS-2013-01).

### 2.1. Animals, Diets, and Experimental Design

#### 2.1.1. Experiment 1 

Eighteen primiparous Dorper × thin-tailed Han crossbred ewes (60.0 ± 1.73 kg body weight (BW)), 12 months of age, were randomly divided, according to the principle of uniform weight, into two dietary treatment groups: a basal diet, or the same basal diet supplemented with TS at 2.0 g/head/day (TS was extracted from Camellia seeds, Xi’an Feida Bio-Tech Co., Ltd., Shanxi, China). The basal diets included pelleted total mixed rations (concentrate) and Chinese wildrye hay (Table 1). For the experimental diet, the TS was mixed with the pelleted concentrate. The ewes were fed 1500 g pelleted concentrate at 800 h and 200 g of Chinese wild rye hay at 1200 h, daily. This feeding level fulfilled the maintenance and growth requirements of yearling ewes (60 kg BW) according to the NRC [17]. All animals were housed in individual pens, had free access to fresh water throughout the experimental period. 

All ewes were moved into metabolism crates after a 14-day adaptation to the diets and another 7-day adaptation to the metabolism crates. The amounts of feed offered, ort, and produced feces were weighed daily and homogenized. A 10% sample was collected during an 8-day collection period, as described by Ma et al. [18]. Urine was collected daily in buckets containing 100 mL of 10% (v/v) H_2_SO_4_. The volume was measured and a sample (10 mL/L of total volume) was collected and stored at −20 °C until analysis. Samples of feed, ort, feces, and urine were pooled to form a composite sample for each ewe.

Ruminal methane production was measured using an open-circuit respirometry system (Sable Systems International, Las Vegas, NV, USA) with three metabolism cages, each fitted with a polycarbonate head box. Measurements of methane production were staggered because only three measurement units were available. On days 0, 2, 4, and 6 of each 8-day collection period, the ewes were moved in sequence from their own metabolism cages to metabolism cages equipped with head boxes for digestibility assays and methane output assessments. After a 24-h adaption period, individual methane production was measured over a 24-h period, as described by Deng et al. [19]. All ewes had been previously trained for confinement in head boxes attached to metabolism cages.

The ewes were weighed when entering and leaving the gas metabolism cages and the average body weight was used as the basis for calculating the metabolic body weight.

#### 2.1.2. Experiment 2

Six ruminally cannulated Dorper × thin-tailed Han crossbred ewes (65.2 ± 2.0 kg BW) were divided into two groups of three, according to a crossover design, and fed one of the following diets: basal diet, or basal diet supplemented with TS (2.0 g/head/day). The composition of the basal diets and the experimental regime were the same as described for Experiment 1. The experiment lasted for 42 days and consisted of two periods lasting 21 days, including 7 days of adaptation. On days 16 and 37, two 50 mL samples of ruminal digesta were collected from the rumen cannula using a syringe attached to a plastic tube (20 mm internal diameter). Samples were collected at 0, 1, 3, 6, and 9 h after the morning feeding for the measurements of ruminal fermentation parameters and microbial flora populations. The pH was measured immediately using a pH meter (Model PB-10, Sartorius Co, Goettingen, Germany). All samples were frozen in liquid nitrogen within 5 min and stored at −80 °C until analysis. 

### 2.2. Analytical Procedures

Dry matter (DM) content was measured by drying samples in a forced-air oven at 135 °C for 2 h (method 930.15; AOAC) [20]. Ash content was measured by placing samples into a muffle furnace at 550 °C for 5 h (method 938.08; AOAC) [20]. Organic matter (OM) was measured as the difference between DM and the ash content. Nitrogen (N) was measured according to the methods of Kjeldahl, using Se as a catalyst. Crude protein (CP) was calculated as 6.25 × N. Gross energy (GE) was measured using a bomb calorimeter (C^200^, IKA Works Inc., Staufen, Germany). Ether extracts (EE) were measured by the weight loss of the DM following extraction with diethyl ether in a Soxhlet extraction apparatus for 8 h (method 920.85; AOAC) [20]. Neutral detergent fibre (NDF) and acid detergent fibre (ADF) were measured according to [21,22]. NDF was measured without a heat stable amylase and expressed inclusive of residual ash. Ruminal volatile fatty acid (VFA) was measured according to the procedure described by Ma et al. [23], and ammonia (NH_3_) was assessed according to Broderick and Kang [24].

The frozen samples were thawed at room temperature, and the total DNA from rumen fluid was extracted using the bead-beating method described by Zhang et al. [25]. The microbial cells were resuspended in a lysis buffer in tubes containing zirconium beads and were bead-beaten at 4600 rpm for 3 min in a mini-bead beater (MM400, Retsch, Hann, Germany), followed by phenol-chloroform extraction [26]. After centrifugation of the sample at 14,000× *g* for 15 min at 4 °C, the supernatant was mixed with a glass milk kit (Gene Clean II Kit, ZZBio Co., Ltd., Shanghai, China) and washed before a final elution step to release the DNA from the glass milk.

Table 2 shows the amplifying primers used for quantitative polymerase chain reaction (qPCR) analysis for microbial flora [27], including total bacteria, methanogens, protozoans, *Fibrobacter succinogenes*, *Ruminococcus flavefaciens*, *Ruminococcus albus*, and *Butyrivibrio fibrisolvens*. All primers were verified using a sequencing and melting curve analysis with a C^1000TM^ Thermal Cycler and bundled software CFX^96^ Manager^TM^ software version 2.1 (Bio-Rad Laboratories, Hercules, CA, USA). The PCR products were purified by gel extraction and ligated into the pGM-T vector (Promega, Madison, WI, USA). The recombinant plasmids were extracted using a plasmid minikit (Omega, Norcross, GA, USA) according to the manufacturer’s instructions and quantified by A260 measurements. Standard curves for microbes were generated with 101–107 copies of recombinant plasmids per μL. The qPCR was performed using SsoFast EvaGreen Supermix (Bio-Rad) on a C^1000TM^ thermal cycler qPCR detection system, with genomic DNA as the template. All PCR amplifications used the following thermal cycling: 95 °C for 10 min, followed by 40 cycles of 94 °C for 20 s, 60 °C for annealing, extension, and collection of fluorescent signals. All samples were prepared from the ewes and each sample was assayed in triplicate.

### 2.3. Statistical Analyses

The data on digestibility and nitrogen balance were analysed using one-way ANOVA. Data for ruminal fermentation parameters and microbial flora measured at each sampling time were analysed using Repeated Measures and Multivariate of General Linear Model. Statistical analyses were performed by using SPSS (SPSS Inc., Chicago, IL, USA). Group differences were considered significant when *p* < 0.05 and tendencies were discussed when 0.05 < *p* < 0.10.

## 3. Results

### 3.1. Nutrient Digestibility

The intake of DM, total tract apparent digestibility of nutrients, and N balance are shown in Table 3. Supplementation with TS increased the apparent digestibility of OM (*p* = 0.001), N (*p* = 0.036), NDF (*p* = 0.001), and ADF (*p* < 0.001) (Table 3). Daily fecal N output decreased from 10.7 to 9.90 g (*p* = 0.036), urinary N decreased from 14.9 to 12.5 g (*p* = 0.001). Overall, the N retention and the ratio of N retention/N intake increased (*p* = 0.001).

### 3.2. Ruminal Fermentation and Methanogenesis

The methane production, ruminal pH, and ruminal concentrations of ammonia and VFA are shown in Table 4. Supplementation of TS did not affect daily methane production by the ewes (*p* > 0.05), but the methane output, scaled to BW^0.75^, decreased from 2.84 to 2.59 (*p* = 0.006) (Table 4). Ruminal pH was similar between the two treatments (*p* = 0.912). TS supplementation decreased ammonia production from 10.7 to 8.3 mmol/L (*p* < 0.001), while total VFA increased from 101.6 to 118.1 mmol/L (*p* = 0.018). The molar proportions of propionate (*p* = 0.007), isobutyrate (*p* = 0.001), butyrate (*p* = 0.002), and isovalerate (*p* = 0.001) were increased by TS supplementation. No difference was observed in the molar proportion of acetate (*p* = 0.171) and valerate (*p* = 0.107). The molar proportion of the ratio of acetate to propionate decreased from 5.23% to 4.50% (*p* = 0.035).

### 3.3. Ruminal Microbial Flora

The effect of TS supplementation on ruminal microbial population is shown in Table 5. Supplementation of TS tended to decrease the population of protozoans (*p* = 0.054) and increased the populations of *F. succinogenes* (*p* = 0.019), whereas population of total bacteria, methanogen, *R. flavefaciens*, *R. albus*, and *B. fibrisolvens* did not change.

## 4. Discussion

### 4.1. Effect of Tea Saponin on Apparent Digestibility and Nitrogen Balance

The effect of TS on nutrient digestibility has been poorly studied. In the present experiment, supplementation with TS increased the apparent digestibility of OM, N, NDF, and ADF. Tea saponins have an important effect on nutrient digestibility [5], whereas saponins from other sources, such as *Quillaja saponaria* or *Yucca schidigera*, are reported to have no effect on diet digestibility [10,28]. The lower N outputs in the urine and feces in the TS group in the present study are consistent with the higher apparent digestibility of dietary N, and may reflect the reduction in protozoan numbers [29]. A related study reported that supplementation with TS improved in vitro OM digestibility [8]. Similarly, Guyader et al. [14] reported a numerical increase in NDF and ADF digestibility in dairy cows; this outcome may be related to the numerically lower dry matter intake (DMI) of lactating dairy cows fed this plant extract, given that a reduction of DMI can be associated with lower rumen filling and greater fiber digestibility. In the present experiment, DMI was not modified in ewes supplemented with TS in DM. Overall, the increase in nutrient digestibility is not related to DMI; it could be explained by the increase in the population of *F. succinogenes*, generally considered the primary organisms responsible for the degradation of plant cell walls in the rumen [30], and by the decrease in the protozoan population.

Nitrogen retention in ruminants has significant benefits for ruminant survival, health, production, and ruminal protection [31]. In the present study, TS supplementation decreased fecal N and urinary N outputs, resulting in a significant N retention. The decrease in urinary N output could be attributed to the decrease in the protozoan population, as previously confirmed by Van Soest [32]. Protozoa contribute to 10–40% of the total rumen nitrogen, so a reduction in this population would mean less predation and lysis of bacteria and, consequently, a lower release of the products of protein breakdown. Jouany [29] assumed that urinary N always decreases with defaunation, due to both the decreased ammonia concentration in the ruminal fluid and the increased capture of urea N for microbial protein synthesis prior to its delivery to the large intestine for recycling in the blood. Koening et al. [33] also reported increases in microbial protein entering the post-digestive tract from the rumen and a promotion of nitrogen utilization. 

### 4.2. Effect of Tea Saponin on Methane Production

In the present study, TS supplementation resulted in an 8.8% decrease in the daily methane emissions, scaled to metabolic BW. TS has been reported to reduce methane production by inhibiting the proliferation of rumen protozoa and perhaps by inhibiting interspecies hydrogen transfer between the protozoa and methanogens, although inhibitory effects on hydrogen-producing bacteria are also possible [34]. Similar results were reported in other studies using TS as a plant extract additive to reduce methane emissions. For example, Guo et al. [12] determined that the mechanism of TS inhibition of methane production involved inhibition of the expression of *mcrA*, a key gene encoding the methyl-coenzyme M reductase enzyme involved in methane synthesis. Hess et al. [35] showed that TS can act directly on methanogens to reduce methane production to levels consistent with those reported by Whitelaw et al. [36] and Dohme et al. [37]. In our study, supplementation with TS had no significant effect on the population of methanogens, but it decreased the population of protozoans. A similar observation was made by Hess et al. [35], who reported a 54% decrease in protozoan counts and a 20% decline in in vitro methane emissions, with no effect on methanogens. These researchers suggested that defaunation reduced methane emissions because of the lower H_2_ supply, which reduced the activity per methanogen. In the present study, TS supplementation increased the molar proportion of propionate (1 mole H_2_ consumed per mole propionate) and decreased the acetate:propionate ratio, indicating a transformation of the rumen from acetate fermentation to propionate fermentation. This switch would lead to a reduction in H_2_ availability for methanogenic archaea [38]. Overall, the observed reduction in methane output, scaled to BW^0.75^, may be related to the size of the protozoan population and the VFA patterns.

### 4.3. Effect of Tea Saponin on Ruminal Fermentation

Supplementation with TS modified the fermentation patterns, resulting in changes in rumen pH, ammonia release, and total VFA content. Ruminal pH is an important index of normal rumen function, and the rumen pH values (pH 5.96–5.98) in the present study were within the normal range for efficient rumen function [39]. Supplementation with TS significantly decreased the ruminal concentration of ammonia but increased the levels of total VFA. Wina et al. [40] suggested that decreases in rumen ammonia concentration were an indirect result of the decreased protozoan numbers caused by addition of TS. Similar to our results, most studies have shown that TS supplementation increased the molar proportion of propionate [13,41,42], although Ramírez-Restrepo et al. [43] reported that butyrate concentration increased and propionate concentration decreased at their highest TS supplementation level. Guyader et al. [44] reported that supplementation with TS decreased the acetate:propionate ratio in an in vitro experiment, in agreement with the results of Hu et al. [9,15] and Guo et al. [12] but a subsequent in vivo experiment in lactating cows showed an increase in the acetate:propionate ratio. Overall, the change in the molar proportions of propionate and butyrate and in the acetate:propionate ratio suggested that TS supplementation could modify rumen fermentation profiles by changing the microbial population or the rate of passage of digesta through the rumen.

### 4.4. Effect of Tea Saponin on Microbial Flora

Supplementation with TS tended to decrease the population of protozoans in the present study, in agreement with the findings of Mao et al. [45] and Zhou et al. [13], who also showed that the rumen protozoan numbers were lower in sheep supplemented with TS. A toxic effect of TS towards protozoa has also been reported previously [9,12,44] in vitro. Wallace et al. [31] indicated that TS might kill or damage protozoa by forming complexes with sterols on the protozoan membrane surface, leading to membrane impairment and eventual disintegration. However, several reports have shown no effect of saponin on protozoa, and some have showed an increase in protozoan numbers [41]. These differences may reflect differences in the experimental diets and the TS dosages. Methanogenic archaea have been observed on the exterior surfaces of rumen protozoa [46]. About 10% to 20% of methanogens live in association with protozoa [47], so a reduction in protozoan numbers would also be expected to reduce methanogen numbers, and thereby decrease methane emissions. However, in vitro [12] and in vivo [45] experiments have shown that TS addition has little effect on the methanogen population, which is consistent with the present findings. Similarly, previous studies indicated that the relative abundance of methanogens in sheep was unaffected by TS supplementation [13,45]. However, the activity of the methanogens could be reduced, as Guo et al. [12] found that TS supplementation inhibited the expression of the *mcrA* gene. 

In the present study, we also used qPCR to quantify four main cellulolytic bacteria and found a selective effect of TS supplementation on rumen bacteria. Unlike the case for *F. succinogenes*, the populations of *R. flavefaciens*, *R. albus*, and *B. fibrisolvens* were unchanged by TS supplementation. Several studies have examined the effects of TS on ruminal microbial flora, but the results have been inconsistent. For example, Guo et al. [12] reported that number of *F. succinogenes* increased significantly with the addition of TS in vitro, in agreement with our results. Conversely, in vivo TS supplementation has been reported to have no effect on the populations of *R. flavefaciens* or *F. succinogenes* [45]. Zhou et al. [13] also reported no changes in the population of *F. succinogenes*. The rumen is a complex system where billions of microbes live, so the effects of TS supplementation on ruminal microbial populations deserve further study.

In order to avoid the adverse effects of stress on entering and leaving the gas metabolism cage, the ewes were weighed twice, before and after the start of the experiment, the average body weight was taken as the basis for the calculation of metabolic body weight. Of course, we considered that body weight may be related to methane emissions, so we used metabolic body weight to eliminate this factor. Converting the average body weight into metabolic body weight is equivalent to unifying the body weight and eliminating the influence of body weight. The relative (per kg metabolic BW) methane emissions are more indicative of differences in methane emissions between different diets.

## 5. Conclusions

In the present study, dietary TS supplementation effectively enhanced OM, N, NDF, and ADF digestibility and reduced daily methane emissions (L/kg BW^0.75^) in ewes. These effects were probably due to decreases in the population of ruminal protozoans and modifications in the VFA profile in response to TS. Further investigation is necessary to explain the mechanisms by which TS exerts these effects on methanogenesis and ruminal microbial flora.

## Figures and Tables

**Table 1:** Ingredients and chemical compositions of experimental diets (% of dry matter (DM)).

Item ^a^	Total Mixed Ration	Chinese Wildrye Hay
Ingredient, % of DM		
Corn	17.0	
Soybean meal	12.0	
Chinese wildrye hay	68.7	
CaHPO_4_	1.35	
Limestone	0.25	
NaCl	0.50	
Premix ^b^	0.24	
Chemical composition (determined)		
DM, (% as fed)	88.6	91.4
OM	80.8	90.6
GE, MJ/kg of DM	17.2	17.6
CP	12.2	8.50
NDF	41.4	70.7
ADF	21.8	38.1

^a^ DM: dry matter; OM: organic matter; GE: gross energy; CP: crude protein; NDF: neutral detergent fibre; ADF: acid detergent fibre. ^b^ Manufactured by Precision Animal Nutrition Research Centre, Beijing, China. The premix contained (per kg): 22.1 g Fe, 2.25 g Cu, 9.82 g Mn, 27.0 g Zn, 0.19 g Se, 0.54 g I, 0.09 g Co, 3.2 g Vitamin A, 0.8 g Vitamin D3, and 0.4 g Vitamin E.

**Table 2:** Primers for qPCR assay.

Target Species	Primer Sequence (5′→3′) ^a^	Amplicon
Total bacteria	F: CGGTGAATACGTTCYCGG	123
	R: GGWTACCTTGTTACGACTT	
Methanogens	F: TTCGGTGGATCDCARAGRGC	140
	R: GBARGTCGWAWCCGTAGAATCC	
Protozoans	F: GCTTTCGWTGGTAGTGTATT	223
	R: CTTGCCCTCYAATCGTWCT	
*Fibrobacter succinogenes*	F: GTTCGGAATTACTGGGCGTAAA	121
	R: CGCCTGCCCCTGAACTATC	
*Ruminococcus flavefaciens*	F: GATGCCGCGTGGAGGAAGAAG	286
	R: CATTTCACCGCTACACCAGGAA	
*Ruminococcus albus*	F: GTTTTAGGATTGTAAACCTCTGTCTT	270
	R: CCTAATATCTACGCATTTCACCGC	
*Butyrivibrio fibrisolvens*	F: TAACATGAGAGTTTGATCCTGGCTC	135
	R: CGTTACTCACCCGTCCGC	

^a^ Primers were designed according to Denman and McSweeney [27].

**Table 3:** Effects of tea saponin (TS) supplementation on the apparent digestibility of nutrients and nitrogen balance in ewes.

Item ^a^	Treatments ^b^		SEM	*p*-Value
	CON	TS		
Apparent digestibility, %				
OM	60.3	66.1	0.99	0.001
N	66.6	69.2	0.63	0.036
NDF	37.9	48.5	1.79	0.001
ADF	35.0	48.3	2.07	<0.001
Fecal N, g/d	10.7	9.90	0.20	0.036
Urinary N, g/d	14.9	12.5	0.42	0.001
N retention, g/d	6.54	9.78	0.56	0.001
N retention/N intake, %	20.3	30.4	1.74	0.001

^a^ DM: dry matter; OM: organic matter; GE: gross energy; CP: crude protein; NDF: neutral detergent fibre; ADF: acid detergent fibre. ^b^ Control (CON) ewes were fed a basal diet; TS ewes were fed the same basal diet supplemented with tea saponin (TS).

**Table 4:** Effects of tea saponin (TS) supplementation on daily methane production and ruminal fermentation in ewes.

Item ^a^	Treatments ^b^		SEM	*p*-Value
	CON	TS		
DM intake, g/d	1512.5	1512.6	0.04	0.593
BW^0.75^, kg	21.4	24.2	0.52	<0.001
Methane production				
L	61.1	62.2	0.77	0.519
L/kg BW^0.75^	2.86	2.57	0.05	0.001
L/kg DMI	40.4	41.1	0.51	0.519
pH	5.98	5.96	0.05	0.912
Ammonia, mmol/L	10.7	8.30	0.33	<0.001
Total VFA, mmol/L	101.6	118.1	3.56	0.018
Molar proportions, %				
Acetate	74.0	80.0	2.17	0.171
Propionate	14.4	18.7	0.82	0.007
Isobutyrate	1.34	2.04	0.12	0.001
Butyrate	9.62	14.2	0.76	0.002
Isovalerate	1.39	2.16	0.13	0.001
Valerate	0.89	1.06	0.05	0.107
Acetate:propionate	5.23	4.50	0.17	0.035

^a^ BW: bodyweight; DMI: dry matter intake; VFA: volatile fatty acids. ^b^ Control (CON) ewes were fed a basal diet; TS ewes were fed the same basal diet supplemented with tea saponin (TS).

**Table 5:** Effects of tea saponin (TS) supplementation on ruminal microbial population.

Microbial Population, per mL of Ruminal Fluid	Treatments ^a^		SEM	*p*-Value
	CON	TS		
Total bacteria, × 10^9^	7.77	8.23	0.40	0.569
Protozoans, × 10^7^	5.44	4.59	0.22	0.054
Methanogens, × 10^7^	7.09	6.18	0.45	0.318
*F. succinogenes*, × 10^5^	4.36	5.41	0.23	0.019
*R. flavefaciens*, × 10^8^	4.06	4.40	0.19	0.372
*R. albus*, × 10^7^	5.30	5.02	0.16	0.385
*B. fibrisolvens*, × 10^8^	6.31	6.49	0.12	0.476

^a^ Control (CON) ewes were fed a basal diet; TS ewes fed the same basal diet supplemented with tea saponin (TS).
